# *In silico* prediction of secretory proteins of *Opisthorchis viverrini, Clonorchis sinensis* and *Fasciola hepatica* that target the host cell nucleus

**DOI:** 10.1016/j.heliyon.2021.e07204

**Published:** 2021-06-05

**Authors:** Claudia Machicado, Maria Pia Soto, Luis Felipe La Chira, Joel Torres, Carlos Mendoza, Luis A. Marcos

**Affiliations:** a Laboratorios de Investigación y Desarrollo, Facultad de Ciencias y Filosofía, Universidad Peruana Cayetano Heredia, Honorio Delgado 430, Lima 31, Peru; b Institute for Biocomputation and Physics of Complex Systems, University of Zaragoza, Spain; c Laboratorio de Investigación en Biología Molecular y Farmacología Experimental, Universidad Católica de Santa María, Urb. San José, San Jose s/n, Arequipa, Peru; d Facultad de Ciencias Biológicas, Universidad Nacional Mayor de San Marcos, Av. Carlos Germán Amezaga 375, Cercado de Lima, Peru; e Facultad de Ciencias Biológicas, Universidad Nacional de Trujillo, Av. Juan Pablo II, Trujillo, 13011, Peru; f Department of Medicine (Division of Infectious Diseases), Department of Microbiology and Immunology, State University of New York at Stony Brook, NY, Stony Brook, USA

**Keywords:** *Opisthorchis viverrini*, *Clonorchis sinensis*, *Fasciola hepatica*, *In silico*, Secretion, Nuclear targeting, Cancer

## Abstract

Liver flukes *Fasciola hepatica*, *Opisthorchis viverrini* and *Clonorchis sinensis* are causing agents of liver and hepatobiliary diseases. A remarkable difference between such worms is the fact that *O. viverrini* and *C. sinensis* are carcinogenic organisms whereas *F. hepatica* is not carcinogenic. The release of secretory factors by carcinogenic flukes seems to contribute to cancer development however if some of these target the host cell nuclei is unknown. We investigated the existence of *O. viverrini and C. sinensis* secretory proteins that target the nucleus of host cells and compared these with the corresponding proteins predicted in *F. hepatica*. Here we applied an algorithm composed by *in silico* approaches that screened and analyzed the potential genes predicted from genomes of liver flukes. We found 31 and 22 secretory proteins that target the nucleus of host cells in *O. viverrini* and *C. sinensis,* respectively, and that have no homologs in *F. hepatica*. These polypeptides have enriched the transcription initiation process and nucleic acid binding in *O. viverrini* and *C. sinensis*, respectively. In addition, other 11 secretory proteins of *O. viverrini* and *C. sinensis*, that target the nucleus of host cells, had *F. hepatica* homologs, have enriched RNA processing function. In conclusion, *O. viverrini* and *C. sinensis* have 31 and 22 genes, respectively, that may be involved in their carcinogenic action through a direct targeting on the host cell nuclei.

## 1 Introduction

Liver infections caused by flukes or trematodes, also termed parasitic flatworms, are considered a serious global public health problem with over 60 million people infected around the world and above 10% population at risk of these infections (Fürst et al., 2012a; Prasad et al., 2011). The burden of these infections in the world is widely distributed with high prevalence rates in Asia and South America (Marcos et al., 2007; Parkinson et al., 2007; Machicado et al., 2016) whereas other regions have less prevalence rates (Saijuntha et al., 2019). This demonstrates the widespread distribution of liver flukes throughout the world that leads to huge economic losses in animal husbandry and morbidity in humans.

Among the causative flukes of trematodiasis, *O. viverrini and C. sinensis,* two human carcinogens, causes opisthorchiasis and clonorchiasis, respectively, that affect both the bile ducts and the liver parenchyma (WHO, 2020). About one out of six individuals with opisthorchiasis may develop cholangiocarcinoma (CCA), or cancer of the bile ducts (Haswell-Elkins et al., 1994; Parkin, 2006). Similarly, chronic infection by *C. sinensis* produces liver fibrosis and CCA. The mechanism of carcinogenesis displayed by these worms is multifactorial and it comprises the mechanical irritation of biliary tissue, the chronic tissue inflammation and the toxic action of secreted factors (Buisson, 2007). Interestingly, secreted mitogens such as Ov-GRN-1 by *O. viverrini* stimulate cell proliferation, angiogenesis and wound repair (Smout et al., 2015). To perform these tasks, the secreted proteins should be either recognized by membrane receptors of host cell or enter the cell. Subcellular targeting will depend on the nature of the parasite proteins. Whether some *O. viverrini* or *C. sinensis* proteins target the nucleus of the host cell is unknown.

*Fasciola hepatica* is a fluke that causes an acute liver disease termed fascioliasis with eosinophilic abscesses through the liver parenchyma and a chronic infection in the biliary ducts leading to fibrosis and sometimes cirrhosis (Marcos et al., 2009). Morbidity caused by fascioliasis in children has been associated with malnutrition and anemia (Cabada and White, 2012). On the other hand, the chronic infection in adults may cause significant morbidity including cholangitis, biliary stones, cholecystitis, biliary obstruction, among other complications (Gandhi et al., 2019; Robinson and Dalton, 2009). Last, but not least, the emergent resistance of *Fasciola* to the only active drug in clinical practice, triclabendazole, both in animals and humans has brought major concerns to the veterinary and medical societies (Overend and Bowen, 1995; Brennan et al., 2007; Kelley et al., 2016).

*O. viverrini, C. sinensis* and *F. hepatica* are relative organisms with close phylogenic relationships and phenotypical features (Fürst et al., 2012b). Despite those biological similarities there is a remarkable difference among liver flukes. *O. viverrini* and *C. sinensis* is a causative agent of cancer whereas *F. hepatica* is not reported as such. Hypothetically, different pathogenicity factors and different host response to each liver fluke infection might suggest that *O. viverrini* and *C. sinensis* releases cancer inducer factors whereas *F. hepatica* might not. The transcriptomes of these flukes might provide insights on these questions and establish differences at a genomic and transcriptomic levels that help explain the carcinogenic properties of *O. viverrini* and *C. sinensis*.

During infection, microorganisms release pathogenic factors and other proteins that facilitate the entry and survival of the pathogen agent. Subcellular targeting of pathogenic effectors to different locations within the host cell would be of vital importance for survival of microorganisms (Eickhoff et al., 2007). A major interest is the nuclear targeting because DNA may be damaged by exogenous molecules. Since DNA damage (i.e. point mutations) is associated with cancer there is an increasing interest in recognizing effectors released by infectious agents, particularly bacteria, that target the host nucleus (Xia et al., 2019). Nuclear targeting displays different mechanisms that depend on the proteins size. Small proteins (MW < 40 KDa) can enter the cell nucleus through passive diffusion. In the other hand, larger proteins (MW > 40KDa) are dependent of a nuclear localization signal (NLS) linked to the immature proteins that establish the final protein location (Freitas and Cunha, 2009). This mechanism has been suggested for the nuclear targeting protein urease A (ureA) of *Helicobacter pylori* that has been associated with the bacterial pathogenicity (Lee et al., 2015).

Some bacterial secretory factors that target host cell nucleus have been identified by *in silico* screening of bacterial genomes aimed to find NLSs. For instance, 49 proteins were predicted to have a putative NLS in *H. pylori* which were further localized in the nucleus by experiments in COS-7 cells (Lee et al., 2012). DNA damage promoted by secretory proteins that target the cell nucleus is a plausible mechanism of cell transformation meaning that carcinogenic agents (i.e. bacteria, parasites and virus) would promote cell transformation through a set of nuclear targeting factors (Benamrouz et al., 2012). For instance, a hypothetical relationship between *Mycoplasma* infection and prostate cancer development has been proposed by the finding of 29 bacterial secretory proteins that target the host cell nucleus (Khan et al., 2016a). Similarly, an *in silico* study predicted 47 secretory and nuclear targeting proteins from *C. pneumoniae* that may have the potential to trigger lung cancer through the alteration in replication, transcription, and DNA damage repair mechanisms (Khan et al., 2016b).

In liver flukes, excretory and secretory products (ESPs) of adult worms have been determined by experimental assays (Mulvenna et al., 2010; Robinson et al., 2009; Di Maggio et al., 2016; Zheng et al., 2011). ESPs from liver flukes are composed by enzymes, cytoskeleton proteins, miRNAs and antioxidants and its composition varies with the developmental stage. The subcellular localization of the ES proteins is mostly cytoplasmic, but some factors are predicted nuclear located (Shi et al., 2020). The fact that extracellular vesicles (EVs), produced by liver flukes, contain a major portion of ESPs suggests that exosomes transport factors that mediate the immune response during the parasite infection (Nawaz et al., 2019). Therefore some nuclear targeting ES proteins released by worms may play a major role in their pathogenesis and further cell transformation by carcinogenic liver flukes. Whether these nuclear ES proteins target or not the host cells is still an open question.

Herein we hypothesize that some ES proteins of both *O. viverrini* and *C. sinensis* target the host nucleus and they are missing in *F. hepatica*. The aim of this study is to predict and compare the nuclear targeting of secretory proteins present in *liver flukes* and to recognize their role within the host cell. Such knowledge will bring insights of unique actions in the host nucleus displayed by factors released by *carcinogenic worms* but unlikely by *F. hepatica* during infection. Future *in vitro* studies of such proteins in *liver flukes* will be needed as well as the determination of their potential effects on the host DNA.

## 2 Materials and methods

### 2.1 Protein database of the parasites genomes

The proteomes deduced from the genomes of *O. viverrini, F. hepatica* and *C. sinensis* were downloaded from the WormBase Parasite database version WBPS9 (https://parasite.wormbase.org/index.html). WormBase Parasite database encompasses flatworms as well as nematodes, and provides genome sequence, genome browsers, semi-automatic annotation and comparative genomics data for approximately one hundred species (Howe et al., 2016, 2017). The *O. viverrini's* genome analyzed had the BioProject ID PRJNA222628, assembly OpiViv1.0 deposited in 2014 (Young et al., 2014). The *F. hepatica* genome was under the BioProject ID PRJEB25283 (Cwiklinski et al., 2015a). The *C. sinensis*' genome analyzed here was under the BioProject ID PRJDA72781 deposited in 2013 (Huang et al., 2013).

### 2.2 Prediction of subcellular localization in eukaryotic cells

The whole proteins coded by genes have a subcellular localization defined as its final location within a cell. Subcellular localization of the whole genes that compose the genomes of *O. viverrini, F. hepatica* and *C. sinensis* was predicted through FUEL-mLoc web-server (http://bioinfo.eie.polyu.edu.hk/FUEL-mLoc/). This algorithm uses Feature-Unified prediction and Explanation of multi-Localization of cellular proteins in multiple organisms (Wan et al., 2017). Those nuclear predicted proteins were selected and analyzed by Balanced Subcellular Localization Predictor, BaCeILo (http://gpcr.biocomp.unibo.it/bacello/pred.htm), a computational tool assists in the prediction of protein subcellular localization including nucleus, cytoplasm, secretory pathway, mitochondrion and chloroplast. BaCeILo is based on different support vector machines organized in a decision tree (Pierleoni et al., 2006). The resulting proteins were named “Nuclear targeting candidates”.

### 2.3 Analysis of physicochemical properties of the nuclear targeting proteins

Theoretical isoelectric point (pI) and molecular weight (MW) were obtained through ProtParam (https://web.expasy.org/protparam/). This tool provides the physicochemical profile for a given protein deposited in Swiss-Prot or TrEMBL or for a user entered protein sequence (Gasteiger et al., 2005). The amino acid sequences were entered in Protparam and data was retrieved for each protein considered as nuclear targeting candidates. Only those proteins with MW less than 40 KDa were selected as potential to target the nucleus of host cells. The resulting proteins were named “Nuclear predicted proteins”.

### 2.4 Gene ontology and recognition of orthologs

Transcript IDs of *O. viverrini* and *C. sinensis* corresponding to the nuclear predicted proteins with <40 KDa were entered in Biomart available in WormBase Parasite Database (https://parasite.wormbase.org/biomart/martview) to obtain the gene description, gene ontology, and UNIPROT IDs. In addition, the section Homology implemented in Biomart was used both to identify homologs between *O. viverrini* and *F. hepatica* as well as *C. sinensis* and *F. hepatica.* First, transcript IDs of *O. viverrini* were entered and then the option “Restrict results to genes with orthologues in *F. hepatica*” was activated, to recognize homologs in these species. Then, transcript IDs of *O. viverrini* were entered and the option “Restrict results to genes without orthologues in *F. hepatica*” to recognize the *O. viverrini* exclusive proteins, not present in *F. hepatica*. The same procedure was applied to identify *C. sinensis* homologs in *F. hepatica* by entering the name of such organisms. Homology analysis was conducted considering the available genomes mentioned in 2.1.

### 2.5 *In silico* secretion analysis

SignalP v 5.0 (Almagro et al., 2019) and SecretomeP v. 2.0 (Bendtsen et al., 2004) were used to predict secretory proteins that belong either to the classical or non-classical secretory pathway, respectively. This analysis was done for Ov-only proteins, Cs-only proteins, Ov-Fh homologs and Cs-Fh homologs. Through SignalP, those proteins that had an N-terminal signal peptide (SP) were considered secretory factors. In SecretomeP, those proteins with a NN-value>0.9 were selected.

### 2.6 Search for genes in available transcriptomes, data from ESPs and extracellular vesicles (EVs) from adult worms

The predicted nuclear ES proteins of *O. viverrini* and *C. sinensis* were searched in data available from their transcriptomes (Young et al., 2014; Huang et al., 2013) as well as in data from their ESPs (Mulvenna et al., 2010; Zheng et al., 2011, 2013; Shi et al., 2020) and EVs, these latter described for *O. viverrini* (Chaiyadet et al., 2015). Data from EVs of *C. sinensis* was not available. Sequences were subjected to either Blastx or Blastp analysis through Blast + against sequences of the available transcriptomes. Those sequences that aligned across >50% of their length and shared more than 40% amino acid identity with p-value<0.05 were considered positive matches. For ESPs and EVs, the polypeptide IDs were searched for through the supplementary data of publications (Mulvenna et al., 2010; Zheng et al., 2011, 2013; Shi et al., 2020; Chaiyadet et al., 2015).

### 2.7 Functional enrichment

The set of genes that resulted unique either to *O. viverrini* or to *C. sinensis* that code nuclear predicted factors, were entered in gProfiler (Reimand et al., 2007) to run an enrichment analysis. The genomes of *O. viverrini* and *C. sinensis*, mentioned in 2.1., were individually selected as the study genomes in gProfiler. Statistical domain scope under the advanced options was set to All known genes/all annotated genes, whereas the Significance threshold was changed to Benjamini-Hochberg FDR and the user threshold set as of 0.05. Graphics and tables were downloaded and further analyzed. The procedure was repeated with both *O. viverrini* genes that had homologs in *F. hepatica* and *C. sinensis* genes that had homologs with *F. hepatica.*

## 3 Results

### 3.1 Prediction of the subcellular localization and physicochemical properties of nuclear predicted proteins

**Figure 1:**
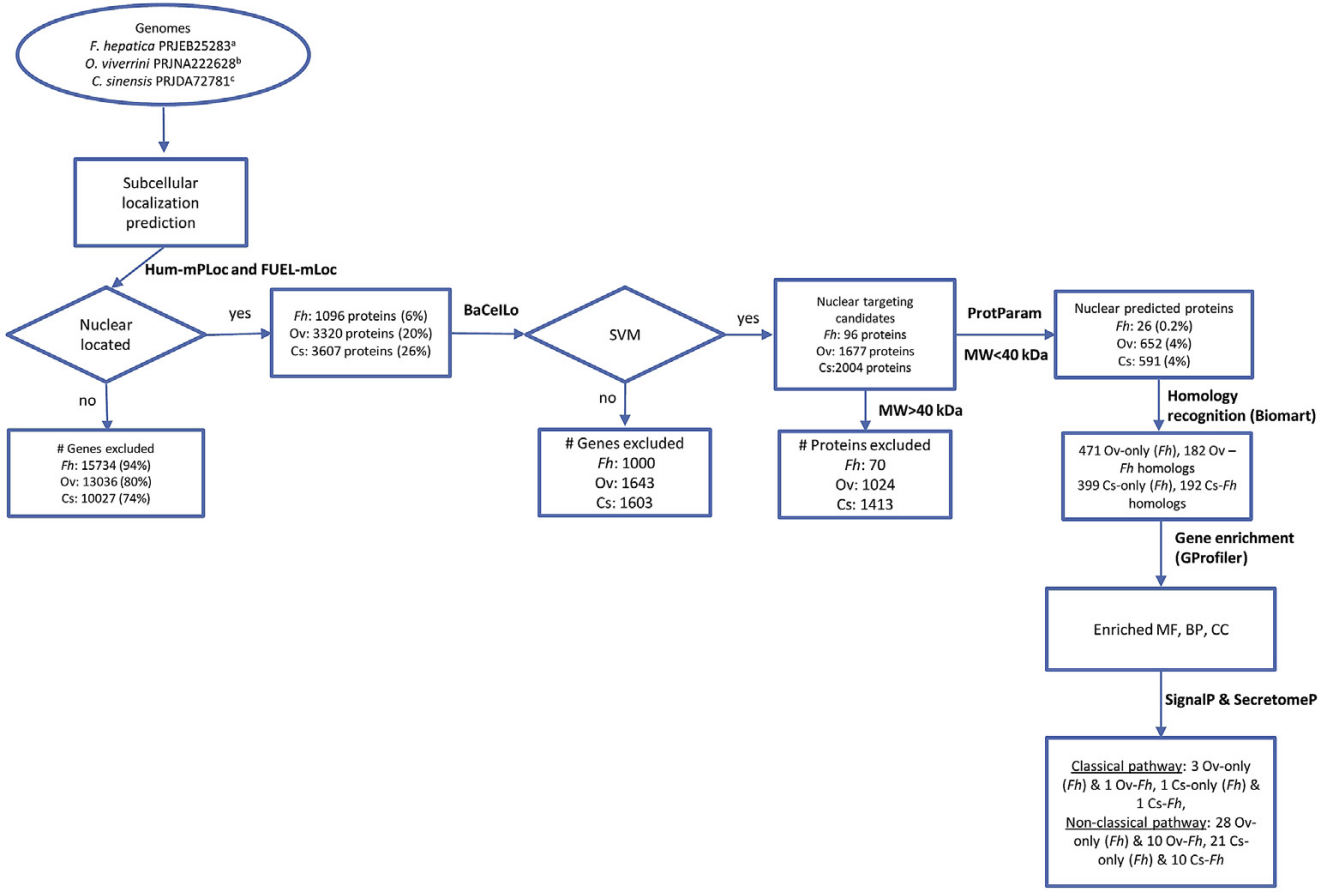
Flowchart of the study. *Fasciola hepatica* (Fh), *Opisthorchis viverrini* (Ov), *Clonorchis sinensis* (Cs). Potential genes predicted from genome: ^a^ n = 16830 genes, ^b^ n = : 16356 genes, ^c^n = 13634 genes. SVM: Support Vector Machine.

*F. hepatica* had more potential genes predicted from the genome (n = 16830) than *O. viverrini* (n = 16356) and *C. sinensis* (n = 13634). The predicted genes of these three parasites were not specific-stage genes which means that these can be expressed in any live stage of liver flukes. Next, these genes were analyzed through various computational tools as shown in Figure 1. First, FUEL-mLoc was applied to recognize nuclear targeting candidates. This tool predicts targeting into 22 different subcellular locations including nucleus, cytoplasm, extracellular, cell membrane, mitochondrion, cytoskeleton, Golgi-apparatus, endoplasmic-reticulum, chloroplast, vacuole, centrosome, lysosome, cell-wall, endosome, peroxisome, synapse, melanosome, spindle-pole-body, microsome, cianelle, undetermined and unknown locations. A total of 3320 polypeptides of *O. viverrini* and 3607 polypeptides of *C. sinensis* were predicted nuclear located which is higher than the number predicted for *F. hepatica* (n = 1096) as shown in Figure 1.

All of these proteins were selected for a second analysis with BaCelLo, to determine subcellular localizations. As a result, *C. sinensis* contained more nuclear targeting candidates (n *=* 2004) than *O. viverrini* (n = 1677) and *F. hepatica (*n = 96) (Figure 1).

The whole predicted nuclear targeting candidates were selected for further analysis. MW and pI were computed for each nuclear targeting candidate (Table S1). In this study those proteins with MW < 40 KDa were selected as candidates to target the cell nucleus according to previous work (Khan et al., 2016a). Our results showed that 39% of *O. viverrini* candidates (n = 652), as well as 29% of *C. sinensis* candidates (n = 591) and 27% of *F. hepatica* candidates (n = 26) had MW < 40 KDa (Figure 1, Table 1). Gene annotations were mostly available for *C. sinensis* and *O. viverrini* candidates than *F. hepatica* proteins (Table 1).

**Table 1:** Nuclear predicted proteins of *O. viverrini*, *C. sinensis* and *F. hepatica* that meet the MW criterion and that were predicted secretory proteins.

Nuclear predicted proteins	Nuclear targeting candidates			Nuclear predicted proteins (MW <40 KDa)			Nuclear predicted Excretion/Secretory (ES) Proteins			
	Ov^a^	Fh^b^	Cs^c^	Ov^a^	Fh^b^	Cs^c^	Ov-only (Fh)	Ov-Fh homologs	Cs-only (Fh)	Cs-Fh homologs
Non annotated	941	65	533	477	17	241	27	4	12	13
Annotated	736	31	1471	175	9	350	4	7	10	9
Total predicted	1677	96	2004	652	26	591	31	11	22	22

a *O. viverrini* Genome Project PRJNA222628.

b *F. hepatica* Genome Project PRJEB25283.

c *C. sinensis* Genome Project PRJDA72781.

### 3.2 Homology recognition and prediction of secretory proteins

To test our hypothesis, we identified through Biomart those nuclear targeting proteins that were unique either to *O. viverrini* or *C. sinensis* and that had no orthologs in *F. hepatica*. These proteins were named Ov-only (Fh) or Cs-only (Fh) proteins, respectively. By applying this criterion, 471 Ov-only (Fh) and 399 Cs-only (Fh) polypeptides were predicted nuclear targeting proteins (Tables 2 and 3). Also we found that 182 and 192 nuclear predicted proteins present in *O. viverrini* and *C. sinensis* had homologs in *F. hepatica,* here termed Ov-Fh and Cs-Fh homologs, respectively (Tables 4 and 6).

**Table 2:** Proteins identified from the *Opisthorchis viverrini* transcriptome that were nuclear predicted ES polypeptides and that were unique to *O. viverrini* (Ov-only).

Ov-only (transcript code)		Secretion pathway		Polypeptide ID	Protein name	pI	MW (kDa)	GO term name			Presence in Transcriptome (Young et al., 2014)	Presence in ESP (Mulvenna et al., 2010)	Presence in EVs (Chaiyadet et al., 2015)
Against Fh	Against Cs	Classical (SignalP)	Non classical (SecretomeP)					MF	BP	CC			
T265_02104		-	+	A0A075AIJ4	Uncharacterized protein	9.33	18.18				Yes	No	No
T265_02161		-	+	A0A075A7P6	Uncharacterized protein	7.80	18.86				Yes	No	No
T265_03674		-	+	A0A075AHE9	Uncharacterized protein	6.71	5.48				Yes	No	No
T265_04711		-	+	A0A074ZM72	Uncharacterized protein	9.98	7.98				Yes	No	No
T265_04717		-	+	A0A074ZMY6	Uncharacterized protein	10.57	16.09				Yes	No	No
T265_04808		-	+	A0A074ZML5	Uncharacterized protein	9.89	10.33				Yes	No	No
T265_06955		-	+	A0A074ZEA4	Uncharacterized protein	9.24	33.15			membrane	Yes	No	No
		-	+							integral to membrane			
T265_07638		-	+	A0A074ZN39	Uncharacterized protein	6.75	27.13				Yes	No	No
T265_12328		-	+	A0A074YTY8	Uncharacterized protein	9.89	17.74				Yes	No	No
T265_15862		-	+	A0A074Z669	Uncharacterized protein	8.84	17.81				Yes	No	No
T265_16081		-	+	A0A074YYX4	Uncharacterized protein	7.98	7.72				Yes	No	No
T265_11103		-	+	A0A074Z480	Uncharacterized protein	6.94	18.64				Yes	No	No
T265_05010		-	+	A0A075AFU9	Uncharacterized protein	5.94	18.90				Yes	No	No
T265_05287		-	+	A0A074ZK83	Uncharacterized protein	9.21	21.17				Yes	No	No
T265_05849		-	+	A0A074ZMN0	Uncharacterized protein	9.97	16.85				Yes	No	No
T265_05881		-	+	A0A075AER1	Uncharacterized protein	10.00	34.11				Yes	No	No
T265_07775		-	+	A0A074ZFZ5	HTH_38 domain-containing protein	10.58	25.89	DNA binding			Yes	No	No
T265_07973		-	+	A0A074ZB32	Uncharacterized protein	8.88	24.28				Yes	No	No
T265_09609		-	+	A0A074Z559	Uncharacterized protein	9.23	33.74				Yes	No	No
T265_10448		-	+	A0A074Z2C0	Uncharacterized protein	9.99	9.62				Yes	No	No
T265_12220		-	+	A0A074YV12	Uncharacterized protein	4.53	16.89				Yes	No	No
T265_13715		-	+	A0A074ZKJ0	Uncharacterized protein	10.39	21.27				Yes	No	No
T265_14284		-	+	A0A074ZCR2	Uncharacterized protein	7.64	36.92	nucleic acid binding			Yes	No	No
T265_11894		+	-	A0A074YXA4	Homeobox domain-containing protein	9.00	27.36	sequence-specific DNA binding	regulation of transcription, DNA-templated	nucleus	Yes	No	No
								DNA binding					
T265_01616		+	-	A0A075AIX5	Uncharacterized protein	6.00	30.82			integral to membrane	Yes	No	No
										membrane			
T265_03703		+	-	A0A074ZRS3	Uncharacterized protein	12.00	22.99				Yes	No	No
T265_00902		-	+	A0A075AJD9	TFIIB-type domain-containing protein	5.68	15.38		transcription from RNA polymerase III promoter	transcription factor TFIIIB complex	Yes	No	No
								metal ion binding	regulation of transcription, DNA-templated				
								core RNA polymerase III binding transcription factor activity	DNA-dependent transcriptional preinitiation complex assembly				
									regulation of transcription from RNA polymerase III promoter				
T265_03631		-	+	A0A074ZQZ9	tRNA (adenine(58)-N(1))-methyltransferase non-catalytic subunit TRM6	8.42	14.15		tRNA methylation	tRNA (m1A) methyltransferase complex	Yes	No	Yes
T265_04852		-	+	A0A075AG04	Uncharacterized protein	9.69	14.76				Yes	No	No
T265_11003		-	+	A0A074ZB31	Uncharacterized protein	7.00	23.38	nucleic acid binding			Yes	No	No
T265_12124		-	+	A0A074Z6I7	Uncharacterized protein	9.84	10.64				Yes	No	No
	T265_14447	-	+	A0A074ZAL8	Uncharacterized protein	7.85	13.02				Yes	No	No
	T265_14603	-	+	A0A074ZDA6	Uncharacterized protein	9.86	36.53				Yes	No	No
	T265_13583	-	+	A0A074ZRH1	Uncharacterized protein	5.73	21.73				Yes	No	No
	T265_01998	-	+	A0A075A868	SEC7 domain-containing protein	6.65	21.31	ARF guanyl-nucleotide exchange factor activity	regulation of ARF protein signal transduction		Yes	No	No
	T265_03266	-	+	A0A074ZT78	Uncharacterized protein	6.55	30.17	DNA binding		nucleus	Yes	No	No
	T265_10781	-	+	A0A074Z5C6	Homeobox domain-containing protein	7.16	32.37	RNA binding		mRNA cap binding complex	Yes	No	No

Gene ontology (GO) obtained through Biomart, MF is Molecular Function, BP is Biological Process and CC is Cellular Component. Polypeptide IDs correspond to the UniProtKB/TrEMBL IDs. The presence and absence of a secretion pathway is denoted with “-” if it is absent and “+” if it is present. References appear in the manuscript.

**Table 3:** Proteins identified from the Clonorchis sinensis transcriptome that were nuclear predicted ES polypeptides and that were unique to *C. sinensis* (Cs-only).

Cs-only transcript code		Secretion pathway		Polypeptide ID	Protein name	pI	MW (kDa)	GO term name			Presence in Transcriptome (Huang et al., 2013)	Presence in ESP (Zheng et al., 2011)	Presence in ESP (Zheng et al., 2013)	Presence in ESP (Shi et al., 2020)
Against Fh	Against Ov	Classical (SignalP)	Non classical (SecretomeP)					MF	BP	CC				
csin100771		-	+	G7Y475	Uncharacterized protein	9.22	18.29				Yes	No	No	No
csin101668		-	+			9.40	36.97				Yes	No	No	No
csin105222		-	+	G7YD84	Endonuclease-reverse transcriptase	9.84	17.87	endonuclease activity	nucleic acid phosphodiester bond hydrolysis		Yes	No	No	No
								RNA-directed DNA polymerase activity	RNA-dependent DNA replication					
csin104730		-	+	G7YC76	Uncharacterized protein	9.38	17.34				Yes	No	No	No
csin103383		-	+	H2KQ76	Zinc finger and BTB domain-containing protein 38	6.42	17.38	nucleic acid binding			Yes	No	No	No
csin110062		-	+	G7YK65	Nuclear hormone receptor family member nhr-8	8.14	17.49	sequence-specific DNA binding	regulation of transcription, DNA-dependent	host cell nucleus	Yes	No	No	No
								sequence-specific DNA binding transcription factor activity		nucleus				
								zinc ion binding						
								DNA binding						
								metal ion binding						
csin111218		-	+	G7YLI0	Uncharacterized protein	8.38	17.37				Yes	No	No	No
csin108410		-	+	G7YI08	Uncharacterized protein	6.59	17.31				Yes	No	No	No
csin110784		-	+	G7YTV7	Pol-related protein	9.84	14.58				Yes	No	No	No
csin111159		-	+	G7YUG2	Uncharacterized protein	9.56	19.00				Yes	No	No	No
csin105509		-	+	G7YDL9	Uncharacterized protein	6.57	33.91				Yes	No	No	No
csin111892		-	+	G7YVI2	C2H2-type domain-containing protein	9.40	30.52	nucleic acid binding			Yes	No	No	No
csin113339		-	+	G7YY80	Histone H3	5.50	22.69	DNA binding		nucleosome	Yes	No	No	No
										protein heterodimerization activity				
										nucleus				
										chromosome				
csin111363		-	+	G7YUQ3	Uncharacterized protein	10.27	29.52				Yes	No	No	No
csin111241		+	-	G7YLJ6	Protein Simiate	8.72	33.82				Yes	No	No	No
csin102657		-	+	H2KPV8	Zinc finger protein 629	9.11	30.06	nucleic acid binding			Yes	No	Yes	No
csin102452		-	+	G7Y7Y9	Peptidyl-prolyl isomerase E (Cyclophilin E)	5.91	25.24	RNA binding			Yes	No	No	No
								nucleic acid binding						
								isomerase activity						
csin104813		-	+	H2KSJ7	La-related protein 6	9.22	31.10				Yes	No	No	No
csin106591		-	+	G7YQ20	Uncharacterized protein	8.63	35.03				Yes	No	No	No
csin104664		-	+	G7YC24	Uncharacterized protein	9.82	29.11				Yes	No	No	No
csin109159		-	+	G7YJ09	Uncharacterized protein	7.06	23.07				Yes	No	No	No
csin110947		-	+	G7YLB1	Uncharacterized protein	8.37	30.49				Yes	No	No	No
	csin103932	-	+	G7YAN4	Myelin transcription factor 1-like protein	7.59	28.11	zinc ion binding	regulation of transcription, DNA-dependent	nucleus	Yes	No	No	No
	csin110299	-	+	G7YTD6	DNA-directed RNA polymerase I subunit H	7.57	18.91	zinc ion binding	mRNA cleavage		Yes	No	No	No
								nucleic acid binding	transcription, DNA-templated					
								metal ion binding						
								DNA-directed RNA polymerase activity						
	csin111481	-	+	G7YLP5	Visual system homeobox 1	9.55	31.10	sequence-specific DNA binding	regulation of transcription, DNA-dependent	nucleus	Yes	No	No	No
								DNA binding						

Gene ontology (GO) obtained through Biomart, MF is Molecular Function, BP is Biological Process and CC is Cellular Component. Polypeptide IDs correspond to the UniProtKB/TrEMBL IDs. The presence and absence of a secretion pathway is denoted with "-" if it is absent and "+" if it is present. References appear in the manuscript.

**Table 4:** Proteins identified from the Opisthorchis viverrini transcriptome that had homologs in *F. hepatica* (Ov-Fh) or *C. sinensis* (Ov-Cs).

Ov homologs transcript code		Secretion pathway		Polypeptide ID	Protein name	pI	MW (kDa)	GO term name		
Ov-Fh	Ov-Cs	Classical (SignalP)	Non classical (SecretomeP)					MF	BP	CC
T265_04509		-	+	A0A074ZZM3	Homeobox domain-containing protein	9.22	28.59	sequence-specific DNA binding	regulation of transcription, DNA-dependent	nucleus
								DNA binding		
T265_09914		-	+	A0A075A372	Cyclin N-terminal domain-containing protein	8.35	39.37			
T265_10276		-	+	A0A074Z2V9	U6 snRNA-associated Sm-like protein LSm1	9.41	24.73	RNA binding	nuclear-transcribed mRNA catabolic process	cytoplasm
								RNA cap binding	mRNA processing	cytoplasmic mRNA processing body
T265_13074		-	+	A0A074ZTW6	Zinc finger, C2H2 type	8.86	32.49	nucleic acid binding		
T265_11866		-	+	A0A074YXE1	Mediator of RNA polymerase II transcription subunit 10	5.29	18.13	transcription cofactor activity	regulation of transcription from RNA polymerase II promoter	mediator complex
										nucleus
T265_15967		-	+	A0A074Z5L5	Uncharacterized protein	5.70	15.74			
T265_00711		+	-	A0A075ABZ8	Uncharacterized protein	5.00	33.60		generation of catalytic spliceosome for second transesterification step	
T265_10781		-	+	A0A074Z5C6	Homeobox domain-containing protein	7.16	32.37	DNA binding		nucleus
T265_01998		-	+	A0A075A868	SEC7 domain-containing protein	6.65	21.31	ARF guanyl-nucleotide exchange factor activity	regulation of ARF protein signal transduction	
T265_03266		-	+	A0A074ZT78	Uncharacterized protein	6.55	30.17			
T265_13583		-	+	A0A074ZRH1	Uncharacterized protein	5.73	21.73			
	T265_11894	+	+	A0A074YXA4	Homeobox domain-containing protein	8.85	27.36			
	T265_00902	-	+	A0A075AJD9	TFIIB-type domain-containing protein	5.68	15.38	core RNA polymerase III binding transcription factor activity	transcription from RNA polymerase III promoter	transcription factor TFIIIB complex
								metal ion binding	DNA-dependent transcriptional preinitiation complex assembly	
									regulation of transcription, DNA-dependent	
	T265_04852	-	+	A0A075AG04	Uncharacterized protein	9.69	14.76			
	T265_06927	-	+	A0A074ZED9	Uncharacterized protein	9.30	17.92			
	T265_03631	-	+	A0A074ZQZ9	tRNA (adenine(58)-N(1))-methyltransferase non-catalytic subunit TRM6	8.42	14.15		tRNA methylation	tRNA (m1A) methyltransferase complex
	T265_11003	-	+	A0A074ZB31	Uncharacterized protein	7.00	23.38	nucleic acid binding		
	T265_12124	-	+	A0A074Z6I7	Uncharacterized protein	9.84	10.64			

Gene ontology (GO) obtained through Biomart, MF is Molecular Function, BP is Biological Process and CC is Cellular Component. Polypeptide IDs correspond to the UniProtKB/TrEMBL IDs. The presence and absence of a secretion pathway is denoted with "-” if it is absent and "+” if it is present. References appear in the manuscript.

Next we applied *in silico* approaches to determine which nuclear predicted proteins were secretory factors, here termed predicted nuclear ES proteins. In summary, 37 Ov-only proteins (missing both in *C. sinensis* nor *F. hepatica*) and 25 Cs-only proteins (missing both in O*. viverrini* and *F. hepatica*) were identified (Tables 2 and 3). Homologies were further recognized among the predicted nuclear ES proteins of the three liver flukes studied. We found that 11 Ov-Fh homologs, 11 Cs-Fh homologs, 13 Ov-Cs homologs and 15 Cs-Ov homologs were predicted secretory and targeting the cell nucleus (Tables 4 and 5). Most of the nuclear predicted ES proteins were recognized by SecretomeP as secretory proteins by the non-classical secretion pathway compared with the classical secretion pathway (Tables 2, 3, 4, and 5). The Ov-only proteins (missing in *C. sinensis* and *F. hepatica*) that were predicted secretory and nuclear targeting had an average MW slightly lower (21 KDa) than Ov-Fh homologs (27 KDa) (Tables 2 and 4). The Ov-only secretory and nuclear proteins had slightly higher average pI (average value = 8) than the Ov-Fh homologs (average value = 7) (Tables 2 and 4). The Cs-only nuclear ES proteins (missing in *O. viverrini* and *F. hepatica*) had identical average MW (25 KDa) and pI (value = 8) to the Cs-Fh homologs (Tables 3 and 5). Also some *O. viverrini* proteins had homologs with *C. sinensis*, and viceversa. Our results showed that the Ov-Cs homologs had a lower average MW (22 KDa) than Cs-Ov homologs (27 KDa) whereas the pI is similar (average value = 8) as shown on Tables 4 and 5. Of interest, no *F. hepatica* nuclear predicted protein was secretory.

**Table 5:** Proteins identified from the Clonorchis sinensis transcriptome that had homologs in *F. hepatica* (Ov-Fh) or *O. viverrini* (Cs-Ov).

Cs homologs transcript code		Secretion pathway		Polypeptide ID	Protein name	pI	MW (kDa)	GO term name		
Cs-Fh	Cs-Ov	Classical (SignalP)	Non classical (SecretomeP)					MF	BP	CC
csin110788		+	-	G7YTV9	Transcription factor HES-4	9.60	37.08	protein dimerization activity		
csin103118		-	+	G7Y944	ETS translocation variant 1/4/5	6.66	29.96	sequence-specific DNA binding	regulation of transcription, DNA-dependent	nucleus
								sequence-specific DNA binding transcription factor activity		
								DNA binding		
csin100942		-	+	G7Y4L2	STARP antigen	11.27	17.01	protein dimerization activity		
csin106523		-	+	G7YQ06	Protein giant	8.17	26.60	sequence-specific DNA binding	developmental process	nucleus
								sequence-specific DNA binding transcription factor activity	regulation of transcription from RNA polymerase II promoter	
									regulation of transcription, DNA-dependent	
csin106380		-	+	G7YF27	Transcription factor SOX1/2/3/14/21	9.83	32.81	DNA binding		nucleus
csin108888		-	+	G7YIQ6	Uncharacterized protein	9.21	23.63			
csin112873		-	+	H2KVQ1	Mediator of RNA polymerase II transcription subunit 10	5.29	18.13	transcription cofactor activity	regulation of transcription from RNA polymerase II promoter	mediator complex
										nucleus
csin109621		-	+	G7YJK7	Homeobox protein MSX-2	10.05	14.71	sequence-specific DNA binding	regulation of transcription, DNA-dependent	nucleus
								DNA binding		
csin103932		-	+	G7YAN4	Myelin transcription factor 1-like protein	7.59	28.11	zinc ion binding	regulation of transcription, DNA-dependent	nucleus
csin110299		-	+	G7YTD6	DNA-directed RNA polymerase I subunit RPA12	7.57	18.91	zinc ion binding	mRNA cleavage	
								nucleic acid binding	transcription, DNA-templated	
								metal ion binding		
								DNA-directed RNA polymerase activity		
csin111481		-	+	G7YLP5	Visual system homeobox 1	9.55	31.10	sequence-specific DNA binding	regulation of transcription, DNA-dependent	nucleus
								DNA binding		
	csin102452	-	+	G7Y7Y9	Peptidyl-prolyl isomerase E (Cyclophilin E)	5.91	25.24	RNA binding		
								nucleic acid binding		
								isomerase activity		
	csin102657	-	+	H2KPV8	Zinc finger protein 629	9.11	30.06	nucleic acid binding		
	csin104813	-	+	G7YCE5	Uncharacterized protein	9.22	36.66			
	csin109159	-	+	G7YJ09	Uncharacterized protein	7.06	23.07			
	csin106591	-	+	G7YQ20	Uncharacterized protein	8.63	35.03			
	csin104664	-	+	G7YC24	Uncharacterized protein	9.82	29.11			
	csin110947	-	+	G7YLB1	Uncharacterized protein	8.37	30.49			

Gene ontology (GO) obtained through Biomart, MF is Molecular Function, BP is Biological Process and CC is Cellular Component. Polypeptide IDs correspond to the UniProtKB/TrEMBL IDs.

The presence and absence of a secretion pathway is denoted with "-” if it is absent and "+” if it is present. References appear in the manuscript.

### 3.3 Search for predicted nuclear ES proteins from *O. viverrini* and *C. sinensis* in experimental data

The predicted nuclear ES proteins of liver flukes were searched for both in the available transcriptomes and ESPs/EVs data obtained from adult flukes. Of the 37 Ov-only proteins (Table 2), all of these appeared in the available transcriptome whereas one is present in EVs (polypeptide ID A0A074ZQZ9), which is missing in *F. hepatica,* and no protein appeared in ESPs (Table 2). According to the ontology data, A0A074ZQZ9 is a tRNA (adenine(58)-N(1))-methyltransferase non-catalytic subunit TRM6 that is theoretically secreted by the non-classical pathway. Additionally, the whole Cs-only proteins (n = 25) appeared in the available transcriptome whereas one Cs-only (Fh), Zinc finger protein 629 (H2KPV8) appeared in ESPs (Table 3).

### 3.3 Gene ontology and enrichment analysis

Gene ontology (GO) was assessed for the 37 *Ov-*only nuclear predicted ES proteins (Table 2). Ontology was available only for 11 Ov polypeptides including five proteins that were missing in *F. hepatica* (A0A074YXA4, A0A075AIX5, A0A075AJD9, A0A074ZQZ9, and A0A074ZB31). DNA binding and regulation of transcription were the most common MF and BP predicted in Ov-only proteins, respectively. In the other hand, both MF and BP were predicted for most of the Ov-Fh homologs and indicated that DNA/RNA binding and regulation of transcription were the most common MF and BP, respectively (Table 4). These findings showed that GO of the Ov-only nuclear predicted ES proteins and Fh-Ov homologs are similar. The same assessment was done to the 25 Cs-only predicted nuclear ES proteins showing that those polypeptides that are missing in *F. hepatica* have DNA/nucleic acid binding and regulation of transcription as main MF and BP, respectively (Table 3). The Cs-Fh homologs had Zn ion- and DNA-binding as main MFs and transcription regulation as main BP (Table 5).

Next, protein enrichment analysis was carried out on the Ov-only (Fh) proteins and Ov-Fh homologs showing that the transcription initiation factor activity is enriched (GO:0006359, adjusted p-value <0.05) and it involved to the polypeptide A0A075AJD9 as shown on Table 6. A0A075AJD9 is an Ov-only (Fh) predicted TFIIB-type domain-containing protein that has a Zinc finger domain. The transcription initiation factor activity was missing among the Ov-Fh homologs. There was no BP or CC obtained from the enrichment analysis for Ov-only (Fh) proteins. Among the 11 Ov-Fh homologs, the RNA cap binding and nucleic acid binding were two enriched MFs (Table 6). The former comprised the U6 snRNA-associated Sm-like protein LSm1 (A0A074Z2V9) whereas the Nucleic acid binding function comprised two Homeobox domain-containing proteins, as well as a Zinc finger, C2H2 type and the U6 snRNA-associated Sm-like protein LSm1. These functions were missing among the Ov-only proteins. Gene expression and mRNA processing were enriched BPs among the Ov-Fh homologs and these involved proteins such as Homeobox domain-containing protein, Mediator of RNA polymerase II transcription subunit 10, and U6 snRNA-associated Sm-like protein LSm1 (Table 6).

**Table 6:** Enrichment analysis obtained for the Ov-only (Fh) nuclear predicted ES proteins and Ov-Fh homologs.

Ov-Fh homologs				MF	Ov-only proteins			
Freq	Polypeptide ID	Protein name	p-adjusted		Freq	Polypeptide ID	Protein name	p-adjusted
Not applicable				RNA polymerase III general transcription initiation factor activity	1	A0A075AJD9	TFIIB-type domain-containing protein	2.269E-02
				general transcription initiation factor activity	1	A0A075AJD9		2.269E-02
1	A0A074Z2V9	U6 snRNA-associated Sm-like protein LSm1	2.516E-02	RNA cap binding	Not applicable			
4	A0A074ZZM3	Homeobox domain-containing protein	2.516E-02	nucleic acid binding				
	A0A074Z2V9	U6 snRNA-associated Sm-like protein LSm1						
	A0A074ZTW6	Zinc finger, C2H2 type						
	A0A074Z5C6	Homeobox domain-containing protein						

Ov-Fh homologs				BP	Ov-only proteins			
Freq	Polypeptide ID	Protein name	p-adjusted		Freq	Polypeptide ID	Protein name	p-adjusted
4	A0A075ABZ8	Uncharacterized protein	1.848E-02	gene expression	Not applicable			
	A0A074ZZM3	Homeobox domain-containing protein						
	A0A074Z2V9	U6 snRNA-associated Sm-like protein LSm1						
	A0A074YXE1	Mediator of RNA polymerase II transcription subunit 10						
4	A0A075ABZ8	Uncharacterized protein	1.848E-02	RNA metabolic process				
	A0A074ZZM3	Homeobox domain-containing protein						
	A0A074Z2V9	U6 snRNA-associated Sm-like protein LSm1						
	A0A074YXE1	Mediator of RNA polymerase II transcription subunit 10						
2	A0A075ABZ8	Uncharacterized protein	1.848E-02	mRNA processing				
	A0A074Z2V9	U6 snRNA-associated Sm-like protein LSm1						
2	A0A075ABZ8	Uncharacterized protein	1.848E-02	mRNA metabolic process				
	A0A074Z2V9	U6 snRNA-associated Sm-like protein LSm1						
3	A0A074ZZM3	Homeobox domain-containing protein	1.848E-02	regulation of metabolic process				
	A0A074Z2V9	U6 snRNA-associated Sm-like protein LSm1						
	A0A074YXE1	Mediator of RNA polymerase II transcription subunit 10						
4	A0A075ABZ8	Uncharacterized protein	1.848E-02	nucleobase-containing compound metabolic process				
	A0A074ZZM3	Homeobox domain-containing protein						
	A0A074Z2V9	U6 snRNA-associated Sm-like protein LSm1						
	A0A074YXE1	Mediator of RNA polymerase II transcription subunit 10						
4	A0A075ABZ8	Uncharacterized protein	1.848E-02	heterocycle metabolic process				
	A0A074ZZM3	Homeobox domain-containing protein						
	A0A074Z2V9	U6 snRNA-associated Sm-like protein LSm1						
	A0A074YXE1	Mediator of RNA polymerase II transcription subunit 10						
1	A0A075ABZ8	Uncharacterized protein	1.848E-02	spliceosomal conformational changes to generate catalytic conformation				
1	A0A075ABZ8	Uncharacterized protein	1.848E-02	generation of catalytic spliceosome for second transesterification step				
4	A0A075ABZ8	Uncharacterized protein	1.848E-02	cellular aromatic compound metabolic process				
	A0A074ZZM3	Homeobox domain-containing protein						
	A0A074Z2V9	U6 snRNA-associated Sm-like protein LSm1						
	A0A074YXE1	Mediator of RNA polymerase II transcription subunit 10						
3	A0A074ZZM3	Homeobox domain-containing protein	1.848E-02	regulation of gene expression				
	A0A074Z2V9	U6 snRNA-associated Sm-like protein LSm1						
	A0A074YXE1	Mediator of RNA polymerase II transcription subunit 10						
3	A0A074ZZM3	Homeobox domain-containing protein	1.848E-02	regulation of macromolecule metabolic process				
	A0A074Z2V9	U6 snRNA-associated Sm-like protein LSm1						
	A0A074YXE1	Mediator of RNA polymerase II transcription subunit 10						
4	A0A075ABZ8	Uncharacterized protein	1.848E-02	nucleic acid metabolic process				
	A0A074ZZM3	Homeobox domain-containing protein						
	A0A074Z2V9	U6 snRNA-associated Sm-like protein LSm1						
	A0A074YXE1	Mediator of RNA polymerase II transcription subunit 10						
4	A0A075ABZ8	Uncharacterized protein	1.848E-02	organic cyclic compound metabolic process				
	A0A074ZZM3	Homeobox domain-containing protein						
	A0A074Z2V9	U6 snRNA-associated Sm-like protein LSm1						
	A0A074YXE1	Mediator of RNA polymerase II transcription subunit 10						
4	A0A074ZZM3	Homeobox domain-containing protein	1.848E-02	regulation of biological process				
	A0A074Z2V9	U6 snRNA-associated Sm-like protein LSm1						
	A0A074YXE1	Mediator of RNA polymerase II transcription subunit 10						
	A0A075A868	SEC7 domain-containing protein						
4	A0A074ZZM3	Homeobox domain-containing protein	2.352E-02	biological regulation				
	A0A074Z2V9	U6 snRNA-associated Sm-like protein LSm1						
	A0A074YXE1	Mediator of RNA polymerase II transcription subunit 10						
	A0A075A868	SEC7 domain-containing protein						
4	A0A075ABZ8	Uncharacterized protein	2.761E-02	cellular nitrogen compound metabolic process				
	A0A074ZZM3	Homeobox domain-containing protein						
	A0A074Z2V9	U6 snRNA-associated Sm-like protein LSm1						
	A0A074YXE1	Mediator of RNA polymerase II transcription subunit 10						
1	A0A075A868	SEC7 domain-containing protein	3.269E-02	regulation of ARF protein signal transduction				
1	A0A075A868	SEC7 domain-containing protein	3.269E-02	ARF protein signal transduction				
1	A0A075A868	SEC7 domain-containing protein	3.758E-02	regulation of Ras protein signal transduction				
2	A0A075ABZ8	Uncharacterized protein	3.758E-02	RNA processing				
	A0A074Z2V9	U6 snRNA-associated Sm-like protein LSm1						
1	A0A075A868	SEC7 domain-containing protein	4.073E-02	Ras protein signal transduction				
1	A0A075A868	SEC7 domain-containing protein	4.194E-02	regulation of small GTPase mediated signal transduction				
1	A0A074Z2V9	U6 snRNA-associated Sm-like protein LSm1	4.591E-02	nuclear-transcribeb mRNA catabolic process				
1	A0A075ABZ8	Uncharacterized protein	4.677E-02	ribonucleoprotein complex subunit organization				
1	A0A075A868	SEC7 domain-containing protein	4.677E-02	regulation of intracellular signal transduction				
2	A0A074ZZM3	Homeobox domain-containing protein	4.677E-02	regulation of nucleic acid-templated transcription				
	A0A074YXE1	Mediator of RNA polymerase II transcription subunit 10						
2	A0A074ZZM3	Homeobox domain-containing protein	4.677E-02	regulation of RNA metabolic process				
	A0A074YXE1	Mediator of RNA polymerase II transcription subunit 10						
2	A0A074ZZM3	Homeobox domain-containing protein	4.677E-02	regulation of biosynthetic process				
	A0A074YXE1	Mediator of RNA polymerase II transcription subunit 10						
1	A0A075ABZ8	Uncharacterized protein	4.677E-02	ribonucleoprotein complex assembly				
2	A0A074ZZM3	Homeobox domain-containing protein	4.677E-02	regulation of transcription, DNA-templated				
	A0A074YXE1	Mediator of RNA polymerase II transcription subunit 10						
2	A0A074ZZM3	Homeobox domain-containing protein	4.677E-02	regulation of nucleobase-containing compound metabolic process				
	A0A074YXE1	Mediator of RNA polymerase II transcription subunit 10						
2	A0A074ZZM3	Homeobox domain-containing protein	4.677E-02	regulation of macromolecule biosynthetic process				
	A0A074YXE1	Mediator of RNA polymerase II transcription subunit 10						
1	A0A074Z2V9	U6 snRNA-associated Sm-like protein LSm1	4.677E-02	mRNA catabolic process				
2	A0A074ZZM3	Homeobox domain-containing protein	4.677E-02	regulation of cellular macromolecula biosynthetic process				
	A0A074YXE1	Mediator of RNA polymerase II transcription subunit 10						
2	A0A074ZZM3	Homeobox domain-containing protein	4.677E-02	regulation of cellular biosynthetic process				
	A0A074YXE1	Mediator of RNA polymerase II transcription subunit 10						
2	A0A074ZZM3	Homeobox domain-containing protein	4.677E-02	regulation of RNA biosynthetic process				
	A0A074YXE1	Mediator of RNA polymerase II transcription subunit 10						
1	A0A074Z2V9	U6 snRNA-associated Sm-like protein LSm1	4.697E-02	RNA catabolic process				

Ov-Fh homologs				CC	Ov-only proteins			
Freq	Polypeptide ID	Protein name	p-adjusted		Freq	Polypeptide ID	Protein name	p-adjusted
Not applicable				transcription factor TFIIIB complex	1	A0A075AJD9	TFIIB-type domain-containing protein	2.521E-02
				tRNA (m1A) methyltransferase complex	1	A0A074ZQZ9	tRNA (adenine(58)-N(1))-methyltransferase non-catalytic subunit TRM6	2.521E-02 2.521E-02
				tRNA methyltransferase complex				
				RNA polymerase III transcription factor complex	1	A0A075AJD9	TFIIB-type domain-containing protein	2.834E-02
1	A0A074Z2V9	U6 snRNA-associated Sm-like protein LSm1	4.030E-03	P-body	Not applicable			
1	A0A074Z2V9	U6 snRNA-associated Sm-like protein LSm1	4.030E-03	ribonucleoprotein granule				
1	A0A074Z2V9	U6 snRNA-associated Sm-like protein LSm1	4.030E-03	cytoplasmic ribonucleoprotein granule				
4	A0A074ZZM3	Homeobox domain-containing protein	2.478E-02	organelle				
	A0A074Z2V9	U6 snRNA-associated Sm-like protein LSm1						
	A0A074YXE1	Mediator of RNA polymerase II transcription subunit 10						
	A0A074Z5C6	Homeobox domain-containing protein						
3	A0A074ZZM3	Homeobox domain-containing protein	2.478E-02	nucleus				
	A0A074YXE1	Mediator of RNA polymerase II transcription subunit 10						
	A0A074Z5C6	Homeobox domain-containing protein						
4	A0A074ZZM3	Homeobox domain-containing protein	2.478E-02	intracellular organelle				
	A0A074Z2V9	U6 snRNA-associated Sm-like protein LSm1						
	A0A074YXE1	Mediator of RNA polymerase II transcription subunit 10						
	A0A074Z5C6	Homeobox domain-containing protein						
4	A0A074ZZM3	Homeobox domain-containing protein	2.922E-02	intracellular anatomical structure				
	A0A074Z2V9	U6 snRNA-associated Sm-like protein LSm1						
	A0A074YXE1	Mediator of RNA polymerase II transcription subunit 10						
	A0A074Z5C6	Homeobox domain-containing protein						
1	A0A074YXE1	Mediator of RNA polymerase II transcription subunit 10	2.922E-02	mediator complex				
3	A0A074ZZM3	Homeobox domain-containing protein	3.715E-02	membrane-bounded organelle				
	A0A074YXE1	Mediator of RNA polymerase II transcription subunit 10						
	A0A074Z5C6	Homeobox domain-containing protein						
3	A0A074ZZM3	Homeobox domain-containing protein	3.715E-02	intracellular membrane-bounded organelle				
	A0A074YXE1	Mediator of RNA polymerase II transcription subunit 10						
	A0A074Z5C6	Homeobox domain-containing protein						

Enrichment analysis done by Gprofiler. MF is Molecular function; BP is Biological process and CC is Cellular component.

The enrichment analysis was also run with the 25 Cs-only (Fh) genes and Cs-Fh homologs (Table 7). The results showed that the nucleic acid binding is an enriched MF that comprised six Cs-only (Fh) genes (GO: 0003676, p-value<0.05) including three zinc finger proteins (H2KPV8, H2KQ76 and G7YVI2) as well as a hormone binding factor, histone 3 and Cyclophilin E (Table 7). One of these factors is Zinc finger protein 629 (H2KPV8), a protein that is present in *C. sinensis* but is missing in *F. hepatica*. Nucleic acid binding was an enriched MF in the group of Cs-Fh homologs but it was regulated by different factors from Cs-only proteins. Among Cs-Fh homologs, nucleic acid binding was mediated by up to seven factors including two homeobox proteins (Homeobox protein MSX-2 and Visual system homeobox 1), DNA-directed RNA polymerase I subunit RPA12, Transcription factor SOX1/2/3/14/21, Protein giant, and ETS translocation variant 1/4/5. Cs-Fh homologs had enriched the transcription regulator activity, protein dimerization and heterocyclic compound binding (Table 7). Enriched BPs associated with Cs-Fh homologs include transcription regulation, RNA biosynthesis, and others and these involved proteins such as ETS translocation variant 1/4/5, Protein giant, Homeobox protein MSX-2, among others (Table 7). There was no BP or CC enriched for Cs-only (Fh) genes.

**Table 7:** Enrichment analysis obtained for the Cs-only (Fh) nuclear predicted ES proteins and Cs-Fh homologs.

Cs-Fh homologs				MF	Cs-only proteins			
Freq	Polypeptide ID	Protein name	p-adjusted		Freq	Polypeptide ID	Protein name	p-adjusted
Not applicable				nucleic acid binding	6	H2KQ76	Zinc finger and BTB domain-containing protein 38	2.126E-02
						G7YK65	Nuclear hormone receptor family member nhr-8	
						G7YVI2	C2H2-type domain-containing protein	
						G7YY80	Histone H3	
						H2KPV8	Zinc finger protein 629	
						G7Y7Y9	Peptidyl-prolyl isomerase E (Cyclophilin E)	
4	G7Y944	ETS translocation variant 1/4/5	6.629E-05	sequence-specific DNA binding	Not applicable			
	G7YQ06	Protein giant						
	G7YJK7	Homeobox protein MSX-2						
	G7YLP5	Visual system homeobox 1						
5	G7Y944	ETS translocation variant 1/4/5	1.750E-04	DNA binding				
	G7YQ06	Protein giant						
	G7YF27	Transcription factor SOX1/2/3/14/21						
	G7YJK7	Homeobox protein MSX-2						
	G7YLP5	Visual system homeobox 1						
3	G7Y944	ETS translocation variant 1/4/5	2.196E-03	transcription regulator activity				
	G7YQ06	Protein giant						
	H2KVQ1	Mediator of RNA polymerase II transcription subunit 10						
6	G7Y944	ETS translocation variant 1/4/5	2.288E-03	nucleic acid binding				
	G7YQ06	Protein giant						
	G7YF27	Transcription factor SOX1/2/3/14/21						
	G7YJK7	Homeobox protein MSX-2						
	G7YTD6	DNA-directed RNA polymerase I subunit RPA12						
	G7YLP5	Visual system homeobox 1						
9	G7YTV9	Transcription factor HES-4	3.952E-03	binding				
	G7Y944	ETS translocation variant 1/4/5						
	G7Y4L2	STARP antigen						
	G7YQ06	Protein giant						
	G7YF27	Transcription factor SOX1/2/3/14/21						
	G7YJK7	Homeobox protein MSX-2						
	G7YAN4	Myelin transcription factor 1-like protein						
	G7YTD6	DNA-directed RNA polymerase I subunit RPA12						
	G7YLP5	Visual system homeobox 1						
10	G7YTV9	Transcription factor HES-4	1.207E-02					
	G7Y944	ETS translocation variant 1/4/5						
	G7Y4L2	STARP antigen						
	G7YQ06	Protein giant						
	G7YF27	Transcription factor SOX1/2/3/14/21						
	H2KVQ1	Mediator of RNA polymerase II transcription subunit 10						
	G7YJK7	Homeobox protein MSX-2						
	G7YAN4	Myelin transcription factor 1-like protein						
	G7YTD6	DNA-directed RNA polymerase I subunit RPA12						
	G7YLP5	Visual system homeobox 1						
2	G7YTV9	Transcription factor HES-4	1.418E-02	protein dimerization activity				
	G7Y4L2	STARP antigen						
2	G7Y944	ETS translocation variant 1/4/5	1.502E-02	DNA-binding transcription factor activity				
	G7YQ06	Protein giant						
6	G7Y944	ETS translocation variant 1/4/5	1.596E-02	heterocyclic compound binding				
	G7YQ06	Protein giant						
	G7YF27	Transcription factor SOX1/2/3/14/21						
	G7YJK7	Homeobox protein MSX-2						
	G7YTD6	DNA-directed RNA polymerase I subunit RPA12						
	G7YLP5	Visual system homeobox 1						
6	G7Y944	ETS translocation variant 1/4/5	1.596E-02	organic cyclic compound binding				
	G7YQ06	Protein giant						
	G7YF27	Transcription factor SOX1/2/3/14/21						
	G7YJK7	Homeobox protein MSX-2						
	G7YTD6	DNA-directed RNA polymerase I subunit RPA12						
	G7YLP5	Visual system homeobox 1						
2	G7YAN4	Myelin transcription factor 1-like protein	3.035E-02	zinc ion binding				
	G7YTD6	DNA-directed RNA polymerase I subunit RPA12						
1	G7YTD6	DNA-directed RNA polymerase I subunit RPA12	3.740E-02	RNA polymerase activity				
2	G7YAN4	Myelin transcription factor 1-like protein	3.740E-02	transition metal ion binding				
	G7YTD6	DNA-directed RNA polymerase I subunit RPA12						
1	G7YTD6	DNA-directed RNA polymerase I subunit RPA12	3.740E-02	5'-3' RNA polymerase activity				
1	G7YTD6	DNA-directed RNA polymerase I subunit RPA12	3.740E-02	DNA-directed 5'-3' RNA polymerase-activity				
1	H2KVQ1	Mediator of RNA polymerase II transcription subunit 10	4.276E-02	transcription coregulator activity				

Cs-Fh homologs				BP	Cs-only proteins			
Freq	Polypeptide ID	Protein name	p-adjusted		Freq	Polypeptide ID	Protein name	p-adjusted
7	G7Y944	ETS translocation variant 1/4/5	6.085E-08	transcription, DNA-templated	Not applicable			
	G7YQ06	Protein giant						
	H2KVQ1	Mediator of RNA polymerase II transcription subunit 10						
	G7YJK7	Homeobox protein MSX-2						
	G7YAN4	Myelin transcription factor 1-like protein						
	G7YTD6	DNA-directed RNA polymerase I subunit RPA12						
	G7YLP5	Visual system homeobox 1						
7	G7Y944	ETS translocation variant 1/4/5	6.085E-08	RNA biosynthetic process				
	G7YQ06	Protein giant						
	H2KVQ1	Mediator of RNA polymerase II transcription subunit 10						
	G7YJK7	Homeobox protein MSX-2						
	G7YAN4	Myelin transcription factor 1-like protein						
	G7YTD6	DNA-directed RNA polymerase I subunit RPA12						
	G7YLP5	Visual system homeobox 1						
7	G7Y944	ETS translocation variant 1/4/5	6.085E-08	nucleic acid-templated transcription				
	G7YQ06	Protein giant						
	H2KVQ1	Mediator of RNA polymerase II transcription subunit 10						
	G7YJK7	Homeobox protein MSX-2						
	G7YAN4	Myelin transcription factor 1-like protein						
	G7YTD6	DNA-directed RNA polymerase I subunit RPA12						
	G7YLP5	Visual system homeobox 1						
6	G7Y944	ETS translocation variant 1/4/5	2.856E-07	regulation of RNA biosynthetic process				
	G7YQ06	Protein giant						
	H2KVQ1	Mediator of RNA polymerase II transcription subunit 10						
	G7YJK7	Homeobox protein MSX-2						
	G7YAN4	Myelin transcription factor 1-like protein						
	G7YLP5	Visual system homeobox 1						
6	G7Y944	ETS translocation variant 1/4/5	2.856E-07	regulation of biosynthetic process				
	G7YQ06	Protein giant						
	H2KVQ1	Mediator of RNA polymerase II transcription subunit 10						
	G7YJK7	Homeobox protein MSX-2						
	G7YAN4	Myelin transcription factor 1-like protein						
	G7YLP5	Visual system homeobox 1						
6	G7Y944	ETS translocation variant 1/4/5	2.856E-07	regulation of cellular macromolecule biosynthetic process				
	G7YQ06	Protein giant						
	H2KVQ1	Mediator of RNA polymerase II transcription subunit 10						
	G7YJK7	Homeobox protein MSX-2						
	G7YAN4	Myelin transcription factor 1-like protein						
	G7YLP5	Visual system homeobox 1						
6	G7Y944	ETS translocation variant 1/4/5	2.856E-07	regulation of transcription, DNA-templated				
	G7YQ06	Protein giant						
	H2KVQ1	Mediator of RNA polymerase II transcription subunit 10						
	G7YJK7	Homeobox protein MSX-2						
	G7YAN4	Myelin transcription factor 1-like protein						
	G7YLP5	Visual system homeobox 1						
6	G7Y944	ETS translocation variant 1/4/5	2.856E-07	regulation of macromolecule biosynthetic process				
	G7YQ06	Protein giant						
	H2KVQ1	Mediator of RNA polymerase II transcription subunit 10						
	G7YJK7	Homeobox protein MSX-2						
	G7YAN4	Myelin transcription factor 1-like protein						
	G7YLP5	Visual system homeobox 1						
7	G7Y944	ETS translocation variant 1/4/5	2.856E-07	heterocycle biosynthetic process				
	G7YQ06	Protein giant						
	H2KVQ1	Mediator of RNA polymerase II transcription subunit 10						
	G7YJK7	Homeobox protein MSX-2						
	G7YAN4	Myelin transcription factor 1-like protein						
	G7YTD6	DNA-directed RNA polymerase I subunit RPA12						
	G7YLP5	Visual system homeobox 1						
6	G7Y944	ETS translocation variant 1/4/5	2.856E-07	regulation of RNA metabolic process				
	G7YQ06	Protein giant						
	H2KVQ1	Mediator of RNA polymerase II transcription subunit 10						
	G7YJK7	Homeobox protein MSX-2						
	G7YAN4	Myelin transcription factor 1-like protein						
	G7YLP5	Visual system homeobox 1						
7	G7Y944	ETS translocation variant 1/4/5	2.856E-07	aromatic compound biosynthetic process				
	G7YQ06	Protein giant						
	H2KVQ1	Mediator of RNA polymerase II transcription subunit 10						
	G7YJK7	Homeobox protein MSX-2						
	G7YAN4	Myelin transcription factor 1-like protein						
	G7YTD6	DNA-directed RNA polymerase I subunit RPA12						
	G7YLP5	Visual system homeobox 1						
6	G7Y944	ETS translocation variant 1/4/5	2.856E-07	regulation of nucleobase-containing compound meta…				
	G7YQ06	Protein giant						
	H2KVQ1	Mediator of RNA polymerase II transcription subunit 10						
	G7YJK7	Homeobox protein MSX-2						
	G7YAN4	Myelin transcription factor 1-like protein						
	G7YLP5	Visual system homeobox 1						
6	G7Y944	ETS translocation variant 1/4/5	2.856E-07	regulation of cellular biosynthetic process				
	G7YQ06	Protein giant						
	H2KVQ1	Mediator of RNA polymerase II transcription subunit 10						
	G7YJK7	Homeobox protein MSX-2						
	G7YAN4	Myelin transcription factor 1-like protein						
	G7YLP5	Visual system homeobox 1						
7	G7Y944	ETS translocation variant 1/4/5	2.856E-07	organic cyclic-compound biosynthetic process				
	G7YQ06	Protein giant						
	H2KVQ1	Mediator of RNA polymerase II transcription subunit 10						
	G7YJK7	Homeobox protein MSX-2						
	G7YAN4	Myelin transcription factor 1-like protein						
	G7YTD6	DNA-directed RNA polymerase I subunit RPA12						
	G7YLP5	Visual system homeobox 1						
7	G7Y944	ETS translocation variant 1/4/5	2.856E-07	nucleobase-containing compound biosynthetic process				
	G7YQ06	Protein giant						
	H2KVQ1	Mediator of RNA polymerase II transcription subunit 10						
	G7YJK7	Homeobox protein MSX-2						
	G7YAN4	Myelin transcription factor 1-like protein						
	G7YTD6	DNA-directed RNA polymerase I subunit RPA12						
	G7YLP5	Visual system homeobox 1						
6	G7Y944	ETS translocation variant 1/4/5	2.856E-07	regulation of nucleic acid-templated transcription				
	G7YQ06	Protein giant						
	H2KVQ1	Mediator of RNA polymerase II transcription subunit 10						
	G7YJK7	Homeobox protein MSX-2						
	G7YAN4	Myelin transcription factor 1-like protein						
	G7YLP5	Visual system homeobox 1						
6	G7Y944	ETS translocation variant 1/4/5	4.205E-07	regulation of gene expression				
	G7YQ06	Protein giant						
	H2KVQ1	Mediator of RNA polymerase II transcription subunit 10						
	G7YJK7	Homeobox protein MSX-2						
	G7YAN4	Myelin transcription factor 1-like protein						
	G7YLP5	Visual system homeobox 1						
7	G7Y944	ETS translocation variant 1/4/5	4.248E-07	RNA metabolic process				
	G7YQ06	Protein giant						
	H2KVQ1	Mediator of RNA polymerase II transcription subunit 10						
	G7YJK7	Homeobox protein MSX-2						
	G7YAN4	Myelin transcription factor 1-like protein						
	G7YTD6	DNA-directed RNA polymerase I subunit RPA12						
	G7YLP5	Visual system homeobox 1						
6	G7Y944	ETS translocation variant 1/4/5	8.088E-07	regulation of nitrogen compound metabolic process				
	G7YQ06	Protein giant						
	H2KVQ1	Mediator of RNA polymerase II transcription subunit 10						
	G7YJK7	Homeobox protein MSX-2						
	G7YAN4	Myelin transcription factor 1-like protein						
	G7YLP5	Visual system homeobox 1						
6	G7Y944	ETS translocation variant 1/4/5	8.088E-07	regulation of primary metabolic process				
	G7YQ06	Protein giant						
	H2KVQ1	Mediator of RNA polymerase II transcription subunit 10						
	G7YJK7	Homeobox protein MSX-2						
	G7YAN4	Myelin transcription factor 1-like protein						
	G7YLP5	Visual system homeobox 1						
6	G7Y944	ETS translocation variant 1/4/5	8.391E-07	regulation of cellular metabolic process				
	G7YQ06	Protein giant						
	H2KVQ1	Mediator of RNA polymerase II transcription subunit 10						
	G7YJK7	Homeobox protein MSX-2						
	G7YAN4	Myelin transcription factor 1-like protein						
	G7YLP5	Visual system homeobox 1						
6	G7Y944	ETS translocation variant 1/4/5	1.056E-06	regulation of macromolecule metabolic process				
	G7YQ06	Protein giant						
	H2KVQ1	Mediator of RNA polymerase II transcription subunit 10						
	G7YJK7	Homeobox protein MSX-2						
	G7YAN4	Myelin transcription factor 1-like protein						
	G7YLP5	Visual system homeobox 1						
6	G7Y944	ETS translocation variant 1/4/5	1.124E-06	regulation of metabolic process				
	G7YQ06	Protein giant						
	H2KVQ1	Mediator of RNA polymerase II transcription subunit 10						
	G7YJK7	Homeobox protein MSX-2						
	G7YAN4	Myelin transcription factor 1-like protein						
	G7YLP5	Visual system homeobox 1						
7	G7Y944	ETS translocation variant 1/4/5	1.351E-06	cellular nitrogen compound biosynthetic process				
	G7YQ06	Protein giant						
	H2KVQ1	Mediator of RNA polymerase II transcription subunit 10						
	G7YJK7	Homeobox protein MSX-2						
	G7YAN4	Myelin transcription factor 1-like protein						
	G7YTD6	DNA-directed RNA polymerase I subunit RPA12						
	G7YLP5	Visual system homeobox 1						
7	G7Y944	ETS translocation variant 1/4/5	1.388E-06	cellular macromolecule biosynthetic process				
	G7YQ06	Protein giant						
	H2KVQ1	Mediator of RNA polymerase II transcription subunit 10						
	G7YJK7	Homeobox protein MSX-2						
	G7YAN4	Myelin transcription factor 1-like protein						
	G7YTD6	DNA-directed RNA polymerase I subunit RPA12						
	G7YLP5	Visual system homeobox 1						
7	G7Y944	ETS translocation variant 1/4/5	1.404E-06	macromolecule biosynthetic process				
	G7YQ06	Protein giant						
	H2KVQ1	Mediator of RNA polymerase II transcription subunit 10						
	G7YJK7	Homeobox protein MSX-2						
	G7YAN4	Myelin transcription factor 1-like protein						
	G7YTD6	DNA-directed RNA polymerase I subunit RPA12						
	G7YLP5	Visual system homeobox 1						
7	G7Y944	ETS translocation variant 1/4/5	1.584E-06	gene expression				
	G7YQ06	Protein giant						
	H2KVQ1	Mediator of RNA polymerase II transcription subunit 10						
	G7YJK7	Homeobox protein MSX-2						
	G7YAN4	Myelin transcription factor 1-like protein						
	G7YTD6	DNA-directed RNA polymerase I subunit RPA12						
	G7YLP5	Visual system homeobox 1						
7	G7Y944	ETS translocation variant 1/4/5	2.716E-06	nucleic acid metabolic process				
	G7YQ06	Protein giant						
	H2KVQ1	Mediator of RNA polymerase II transcription subunit 10						
	G7YJK7	Homeobox protein MSX-2						
	G7YAN4	Myelin transcription factor 1-like protein						
	G7YTD6	DNA-directed RNA polymerase I subunit RPA12						
	G7YLP5	Visual system homeobox 1						
7	G7Y944	ETS translocation variant 1/4/5	5.173E-06	cellular biosynthetic process				
	G7YQ06	Protein giant						
	H2KVQ1	Mediator of RNA polymerase II transcription subunit 10						
	G7YJK7	Homeobox protein MSX-2						
	G7YAN4	Myelin transcription factor 1-like protein						
	G7YTD6	DNA-directed RNA polymerase I subunit RPA12						
	G7YLP5	Visual system homeobox 1						
7	G7Y944	ETS translocation variant 1/4/5	5.644E-06	organic substance biosynthetic process				
	G7YQ06	Protein giant						
	H2KVQ1	Mediator of RNA polymerase II transcription subunit 10						
	G7YJK7	Homeobox protein MSX-2						
	G7YAN4	Myelin transcription factor 1-like protein						
	G7YTD6	DNA-directed RNA polymerase I subunit RPA12						
	G7YLP5	Visual system homeobox 1						
7	G7Y944	ETS translocation variant 1/4/5	6.273E-06	biosynthetic process				
	G7YQ06	Protein giant						
	H2KVQ1	Mediator of RNA polymerase II transcription subunit 10						
	G7YJK7	Homeobox protein MSX-2						
	G7YAN4	Myelin transcription factor 1-like protein						
	G7YTD6	DNA-directed RNA polymerase I subunit RPA12						
	G7YLP5	Visual system homeobox 1						
7	G7Y944	ETS translocation variant 1/4/5	6.569E-06	nucleobase-containing compound metabolic process				
	G7YQ06	Protein giant						
	H2KVQ1	Mediator of RNA polymerase II transcription subunit 10						
	G7YJK7	Homeobox protein MSX-2						
	G7YAN4	Myelin transcription factor 1-like protein						
	G7YTD6	DNA-directed RNA polymerase I subunit RPA12						
	G7YLP5	Visual system homeobox 1						
7	G7Y944	ETS translocation variant 1/4/5	7.668E-06	heterocycle metabolic process				
	G7YQ06	Protein giant						
	H2KVQ1	Mediator of RNA polymerase II transcription subunit 10						
	G7YJK7	Homeobox protein MSX-2						
	G7YAN4	Myelin transcription factor 1-like protein						
	G7YTD6	DNA-directed RNA polymerase I subunit RPA12						
	G7YLP5	Visual system homeobox 1						
7	G7Y944	ETS translocation variant 1/4/5	7.668E-06	cellular aromatic compound metabolic process				
	G7YQ06	Protein giant						
	H2KVQ1	Mediator of RNA polymerase II transcription subunit 10						
	G7YJK7	Homeobox protein MSX-2						
	G7YAN4	Myelin transcription factor 1-like protein						
	G7YTD6	DNA-directed RNA polymerase I subunit RPA12						
	G7YLP5	Visual system homeobox 1						
7	G7Y944	ETS translocation variant 1/4/5	7.975E-06	organic cyclic compound metabolic process				
	G7YQ06	Protein giant						
	H2KVQ1	Mediator of RNA polymerase II transcription subunit 10						
	G7YJK7	Homeobox protein MSX-2						
	G7YAN4	Myelin transcription factor 1-like protein						
	G7YTD6	DNA-directed RNA polymerase I subunit RPA12						
	G7YLP5	Visual system homeobox 1						
7	G7Y944	ETS translocation variant 1/4/5	2.202E-05	cellular nitrogen compound metabolic process				
	G7YQ06	Protein giant						
	H2KVQ1	Mediator of RNA polymerase II transcription subunit 10						
	G7YJK7	Homeobox protein MSX-2						
	G7YAN4	Myelin transcription factor 1-like protein						
	G7YTD6	DNA-directed RNA polymerase I subunit RPA12						
	G7YLP5	Visual system homeobox 1						
6	G7Y944	ETS translocation variant 1/4/5	7.729E-05	regulation of cellular process				
	G7YQ06	Protein giant						
	H2KVQ1	Mediator of RNA polymerase II transcription subunit 10						
	G7YJK7	Homeobox protein MSX-2						
	G7YAN4	Myelin transcription factor 1-like protein						
	G7YLP5	Visual system homeobox 1						
6	G7Y944	ETS translocation variant 1/4/5	8.898E-05	regulation of biological process				
	G7YQ06	Protein giant						
	H2KVQ1	Mediator of RNA polymerase II transcription subunit 10						
	G7YJK7	Homeobox protein MSX-2						
	G7YAN4	Myelin transcription factor 1-like protein						
	G7YLP5	Visual system homeobox 1						
7	G7Y944	ETS translocation variant 1/4/5	1.048E-04	cellular macromolecule metabolic process				
	G7YQ06	Protein giant						
	H2KVQ1	Mediator of RNA polymerase II transcription subunit 10						
	G7YJK7	Homeobox protein MSX-2						
	G7YAN4	Myelin transcription factor 1-like protein						
	G7YTD6	DNA-directed RNA polymerase I subunit RPA12						
	G7YLP5	Visual system homeobox 1						
6	G7Y944	ETS translocation variant 1/4/5	1.316E-04	biological regulation				
	G7YQ06	Protein giant						
	H2KVQ1	Mediator of RNA polymerase II transcription subunit 10						
	G7YJK7	Homeobox protein MSX-2						
	G7YAN4	Myelin transcription factor 1-like protein						
	G7YLP5	Visual system homeobox 1						
7	G7Y944	ETS translocation variant 1/4/5	4.436E-04	macromolecule metabolic process				
	G7YQ06	Protein giant						
	H2KVQ1	Mediator of RNA polymerase II transcription subunit 10						
	G7YJK7	Homeobox protein MSX-2						
	G7YAN4	Myelin transcription factor 1-like protein						
	G7YTD6	DNA-directed RNA polymerase I subunit RPA12						
	G7YLP5	Visual system homeobox 1						
7	G7Y944	ETS translocation variant 1/4/5	8.093E-04	nitrogen compound metabolic process				
	G7YQ06	Protein giant						
	H2KVQ1	Mediator of RNA polymerase II transcription subunit 10						
	G7YJK7	Homeobox protein MSX-2						
	G7YAN4	Myelin transcription factor 1-like protein						
	G7YTD6	DNA-directed RNA polymerase I subunit RPA12						
	G7YLP5	Visual system homeobox 1						
2	G7YQ06	Protein giant	1.098E-03	regulation of transcription by RNA polymerase II				
	H2KVQ1	Mediator of RNA polymerase II transcription subunit 10						
7	G7Y944	ETS translocation variant 1/4/5	1.113E-03	cellular metabolic process				
	G7YQ06	Protein giant						
	H2KVQ1	Mediator of RNA polymerase II transcription subunit 10						
	G7YJK7	Homeobox protein MSX-2						
	G7YAN4	Myelin transcription factor 1-like protein						
	G7YTD6	DNA-directed RNA polymerase I subunit RPA12						
	G7YLP5	Visual system homeobox 1						
7	G7Y944	ETS translocation variant 1/4/5	1.123E-03	primary metabolic process				
	G7YQ06	Protein giant						
	H2KVQ1	Mediator of RNA polymerase II transcription subunit 10						
	G7YJK7	Homeobox protein MSX-2						
	G7YAN4	Myelin transcription factor 1-like protein						
	G7YTD6	DNA-directed RNA polymerase I subunit RPA12						
	G7YLP5	Visual system homeobox 1						
7	G7Y944	ETS translocation variant 1/4/5	1.378E-03	organic substance metabolic process				
	G7YQ06	Protein giant						
	H2KVQ1	Mediator of RNA polymerase II transcription subunit 10						
	G7YJK7	Homeobox protein MSX-2						
	G7YAN4	Myelin transcription factor 1-like protein						
	G7YTD6	DNA-directed RNA polymerase I subunit RPA12						
	G7YLP5	Visual system homeobox 1						
2	G7YQ06	Protein giant	2.063E-03	transcription by RNA polymerase II				
	H2KVQ1	Mediator of RNA polymerase II transcription subunit 10						
1	G7YTD6	DNA-directed RNA polymerase I subunit RPA12	2.771E-03	mRNA cleavage				
7	G7Y944	ETS translocation variant 1/4/5	3.462E-03	metabolic process				
	G7YQ06	Protein giant						
	H2KVQ1	Mediator of RNA polymerase II transcription subunit 10						
	G7YJK7	Homeobox protein MSX-2						
	G7YAN4	Myelin transcription factor 1-like protein						
	G7YTD6	DNA-directed RNA polymerase I subunit RPA12						
	G7YLP5	Visual system homeobox 1						
7	G7Y944	ETS translocation variant 1/4/5	2.023E-02	cellular process				
	G7YQ06	Protein giant						
	H2KVQ1	Mediator of RNA polymerase II transcription subunit 10						
	G7YJK7	Homeobox protein MSX-2						
	G7YAN4	Myelin transcription factor 1-like protein						
	G7YTD6	DNA-directed RNA polymerase I subunit RPA12						
	G7YLP5	Visual system homeobox 1						
1	G7YTD6	DNA-directed RNA polymerase I subunit RPA12	2.583E-02	RNA phosphodiester bond hydrolysis				
7	G7Y944	ETS translocation variant 1/4/5	4.263E-02	biological process				
	G7YQ06	Protein giant						
	H2KVQ1	Mediator of RNA polymerase II transcription subunit 10						
	G7YJK7	Homeobox protein MSX-2						
	G7YAN4	Myelin transcription factor 1-like protein						
	G7YTD6	DNA-directed RNA polymerase I subunit RPA12						
	G7YLP5	Visual system homeobox 1						

Cs-Fh homologs				CC	Cs-only proteins			
Freq	Polypeptide ID	Protein name	p-adjusted		Freq	Polypeptide ID	Protein name	p-adjusted
7	G7Y944	ETS translocation variant 1/4/5	1.151E-06	nucleus	Not applicable			
	G7YQ06	Protein giant						
	G7YF27	Transcription factor SOX1/2/3/14/21						
	H2KVQ1	Mediator of RNA polymerase II transcription subunit 10						
	G7YJK7	Homeobox protein MSX-2						
	G7YAN4	Myelin transcription factor 1-like protein						
	G7YLP5	Visual system homeobox 1						
7	G7Y944	ETS translocation variant 1/4/5	7.056E-06	membrane-bounded organelle				
	G7YQ06	Protein giant						
	G7YF27	Transcription factor SOX1/2/3/14/21						
	H2KVQ1	Mediator of RNA polymerase II transcription subunit 10						
	G7YJK7	Homeobox protein MSX-2						
	G7YAN4	Myelin transcription factor 1-like protein						
	G7YLP5	Visual system homeobox 1						
7	G7Y944	ETS translocation variant 1/4/5	7.056E-06	intracellular membrane-bounded organelle				
	G7YQ06	Protein giant						
	G7YF27	Transcription factor SOX1/2/3/14/21						
	H2KVQ1	Mediator of RNA polymerase II transcription subunit 10						
	G7YJK7	Homeobox protein MSX-2						
	G7YAN4	Myelin transcription factor 1-like protein						
	G7YLP5	Visual system homeobox 1						
7	G7Y944	ETS translocation variant 1/4/5	4.771E-05	intracellular organelle				
	G7YQ06	Protein giant						
	G7YF27	Transcription factor SOX1/2/3/14/21						
	H2KVQ1	Mediator of RNA polymerase II transcription subunit 10						
	G7YJK7	Homeobox protein MSX-2						
	G7YAN4	Myelin transcription factor 1-like protein						
	G7YLP5	Visual system homeobox 1						
7	G7Y944	ETS translocation variant 1/4/5	5.316E-05	organelle				
	G7YQ06	Protein giant						
	G7YF27	Transcription factor SOX1/2/3/14/21						
	H2KVQ1	Mediator of RNA polymerase II transcription subunit 10						
	G7YJK7	Homeobox protein MSX-2						
	G7YAN4	Myelin transcription factor 1-like protein						
	G7YLP5	Visual system homeobox 1						
7	G7Y944	ETS translocation variant 1/4/5	9.884E-05	intracellular anatomical structure				
	G7YQ06	Protein giant						
	G7YF27	Transcription factor SOX1/2/3/14/21						
	H2KVQ1	Mediator of RNA polymerase II transcription subunit 10						
	G7YJK7	Homeobox protein MSX-2						
	G7YAN4	Myelin transcription factor 1-like protein						
	G7YLP5	Visual system homeobox 1						
7	G7Y944	ETS translocation variant 1/4/5	1.816E-02	cellular anatomical entity				
	G7YQ06	Protein giant						
	G7YF27	Transcription factor SOX1/2/3/14/21						
	H2KVQ1	Mediator of RNA polymerase II transcription subunit 10						
	G7YJK7	Homeobox protein MSX-2						
	G7YAN4	Myelin transcription factor 1-like protein						
	G7YLP5	Visual system homeobox 1						
1	H2KVQ1	Mediator of RNA polymerase II transcription subunit 10	1.816E-02	mediator complex				
7	G7Y944	ETS translocation variant 1/4/5	1.816E-02	cellular component				
	G7YQ06	Protein giant						
	G7YF27	Transcription factor SOX1/2/3/14/21						
	H2KVQ1	Mediator of RNA polymerase II transcription subunit 10						
	G7YJK7	Homeobox protein MSX-2						
	G7YAN4	Myelin transcription factor 1-like protein						
	G7YLP5	Visual system homeobox 1						

Enrichment analysis done by Gprofiler. MF is Molecular function; BP is Biological process and CC is Cellular component.

In summary, the transcription activity was a MF strongly associated with at least one Ov-only (Fh) protein whereas such activity is missing among the Ov-Fh homologs (Table 6). RNA processing was a BP enriched in the Ov-Fh homologs but it was missing in the Ov-only proteins (Table 6). At the contrary, Cs-only (Fh) proteins and Cs-Fh homologs had enriched the acid nucleic binding function through different factors that regulate such activity.

## 4 Discussion

In this study we interrogated the entire predicted genes from genomes of *O. viverrini, C. sinensis* and *F. hepatica* to look for secretory proteins that target the nuclei of host cells. Our main interest was to identify proteins unique to carcinogenic liver flukes and missing in *F. hepatica*, to learn about their associated functions. We applied both MpLoc and BaCelLo, two *in silico* machines for subcellular localization and recognition of nuclear localization, followed by an additional criterion related to the protein size. Our rationale was that the property of proteins to passively cross into host subcellular compartments is governed by their molecular weight (Tran and Wente, 2006). Therefore, we established that nuclear targeting candidates with molecular weight below 40 KDa were able to passively cross the nucleus, as it was previously described (Khan, 2014). This method has demonstrated to be a suitable tool as an initial exploration for nuclear targeting prediction in *E. coli, M. hominis* and *C. pneumoniae* (Khan, 2014; Khan et al., 2016a, 2016b).

As a first and notable finding was the number of genes encoding nuclear predicted proteins of *F. hepatica* that is notably lower than these predicted in *O. viverrini* and *C. sinensis*. According to our results, the carcinogenic helminths have thousands of nuclear predicted proteins whereas *F. hepatica* have only 26. This amount is comparable with the number of nuclear predicted proteins in bacteria, such as *H. pylori* (n = 26), *M. hominis* (n = 29) and *C. pneumoniae* (n = 47) (Lee et al., 2012; Khan et al., 2016a, 2016b).

The transcriptomes of liver flukes have been sequenced and analyzed and the existence of genes encoding peptidases, cathepsins, metabolic enzymes and transporters is particularly relevant in this group of worms (Cwiklinski et al., 2015a; Young et al., 2014; Huang et al., 2013). Although the subcellular localization of proteins may be estimated from the transcriptomes of liver flukes, it is the first time to the best of our knowledge that the secretory proteins that target the nucleus of host cells are identified in these three related flukes through *in silico* approaches. Here by applying a homology search we found that some genes are present in the carcinogenic liver flukes but are missing in *F. hepatica*, here termed Ov-only (Fh) and Cs-only (Fh) genes. We predicted that a total of 471 and 399 nuclear targeting proteins are present only either in *O. viverrini* or *C. sinensis*, respectively, but these are missing in *F. hepatica*. Such polypeptides, that are not specific-stage factors, may be associated with some unique features shown in infection by O*. viverrini* and *C. sinensis.* In addition, we predicted that carcinogenic liver flukes have homologs in *F. hepatica*, here termed Ov-Fh and Cs-Fh homologs. We found that 182 and 192 nuclear predicted proteins of *O. viverrini* and *C. sinensis*, respectively, had homologs in *F. hepatica*. Those factors may be associated with common features of the pathogenesis of liver flukes infection.

Part of the transcriptome of liver flukes is composed by genes encoding excretory-secretory (ES) proteins. ESPs from liver flukes contain ES proteins that are a group of polypeptides that are excreted to the extracellular medium where they mediate host-pathogen interactions (Suttiprapa et al., 2018). The secretomes of liver flukes have been previously predicted from the corresponding transcriptomes and most recently determined by experimental techniques. The available secretomes varies across the worms where *O. viverrini* has the biggest secretomes (n = 300) followed by *F. hepatica* (n = 202) and *C. sinensis* (n = 175) (Mulvenna et al., 2010; Di Maggio et al., 2016; Shi et al., 2020). Given that we aimed to predict the secretory proteins that target the nuclei of host cells, the whole nuclear predicted proteins were tested to identify which ones are secreted to the extracellular environment. We applied two approaches including SignalP v 5.0 (Almagro et al., 2019) and SecretomeP v. 2.0 (Bendtsen et al., 2004) which were previously utilized to predict secretory proteins in *Toxoplasma gondii* (Syn et al., 2018). Our results showed the existence of 31 Ov-only (Fh) proteins that have the transcription initiation activity enriched, involving a predicted TFIIB-type domain-containing protein (A0A075AJD9). Zinc finger TFIIB-type proteins assists the RNA polymerase II in the promoter recognition during the transcription. TFIIB-type domain-containing protein from *O. viverrini* is predicted secretory and it targets the host cell nucleus which suggests a relevant strategy of this fluke to interfere with the normal transcription of the host cell. Eukaryotic RNA polymerases are highly conserved and have identical substrates. Therefore a competitive mechanism between the parasites' and human's TFIIB-type domain-containing protein may lead to abnormal transcription (Papatpremsiri et al., 2015; Gasser et al., 2017). Given that the polypeptide A0A075AJD9 had no homologs in *F. hepatica* and it was predicted to be secretory and nuclear targeted, we hypothesize that such protein may be involved in the carcinogenic mechanism displayed by *O. viverrini*. However the polypeptide A0A075AJD9 is missing in the available data from the ESPs and EV cargo (Mulvenna et al., 2010; Chaiyadet et al., 2015). Most proteins contained within *O. viverrini* ESPs are associated with enzyme activity and cytoskeleton with less frequency of nuclear proteins (Mulvenna et al., 2010). According to our results, the existence of the TFIIB-type domain-containing protein and its hypothetical role in the opisthorchiasis and cancer development should be further studied. In addition, we found that the polypeptide A0A074ZQZ9, an Ov-only (Fh) found in EVs, is one out of the 108 proteins contained in *O. viverrini* EVs that were demonstrated to promote cell transformation (Chaiyadet et al., 2015). This latter has been mostly associated with the action of granulin and thioredoxin, both present in ESPs, which induced proliferation of host cells by *in vitro* assays (Mulvenna et al., 2010, Chaiyadet et al., 2015). The involvement of a nuclear targeting proteins has not been investigated but our results suggests that tRNA (adenine(58)-N(1))-methyltransferase non-catalytic subunit TRM6 (A0A074ZQZ9) may have an effect on the tRNA methylation of host cells. tRNA methylation and its role in infection by liver flukes is currently an unknown topic.

On the other hand, we found that *C. sinensis* has 22 nuclear predicted ES genes that are missing in *F. hepatica* (Cs-only proteins). Such genes are transcribed and one gene encoding Zinc finger protein 629 is among the ESPs previously characterized in *C. sinensis* (Zheng et al., 2011, 2013; Shi et al., 2020). The role of ESPs in the pathogenesis of clonorchiasis is still unclear but some antigenic factors such as Cs-FBPase, CsMAP-2 and CsAP have been characterized (Zheng et al., 2011, 2013). Zinc finger protein 629 secreted by *C. sinensis* (and missing in *F. hepatica*) has not a demonstrated function but its human homolog Zinc finger protein 423 is an oncogene that contributes to the development of CCA (Chaiprasert et al., 2019). The function of Zinc finger protein 629 needs to be further investigated.

The finding that 11 polypeptides either in *O. viverrini* or *C. sinensis* are nuclear predicted ES and have homologs in *F. hepatica* (Ov-Fh or Cs-Fh homologs) shows that these phylogenetically related organisms display equivalent mechanisms to manipulate essential activities in the host nucleus. According to the enrichment analysis of Ov-Fh homologs, those common polypeptides are involved in RNA processing and spliceosome function. Consequently, the mRNA maturation in the host cells may be disrupted by the presence of exogenous parasites factors released during the infection by *O. viverrini* and *F. hepatica*. According to our results on Cs-Fh homologs, various activities including heterocyclic compound binding, transcription regulator activity and DNA binding are commonly present in *C. sinensis* and *F. hepatica.* Given that such factors were found in both flukes, these proteins are not expected to be associated with *O. viverrini/C. sinensis* tumorigenesis.

In our study *F. hepatica* had no predicted nuclear ES protein which constitutes a major difference with the carcinogenic liver flukes. ES proteins of *F. hepatica* mainly include proteases, proteases inhibitors and detoxifying enzymes but nuclear proteins have not been described (Di Maggio et al., 2016). A group of ES proteins of *F. hepatica* promote the production of cytokines by the host such as IL2, IL-7 and IFN-γ that participate in modulating host immune response (Liu et al., 2017). Again, the existence of nuclear targeting within ES proteins of *F. hepatica* has not been previously investigated but our results suggest that such a type of proteins is lacking in the *F. hepatica* proteome.

The ES proteins have been characterized for liver flukes and these vary across worms. For instance, ES proteins of *O. viverrini* include peptidases, heat shock proteins and superoxide dismutase whereas lipid-binding and -transport factors, cysteine-type peptidase and peptidase inhibitor have been characterized in *C. sinensis* (Young et al., 2014; Huang et al., 2013). ES proteins from *F. hepatica* mainly include peptidases and cytokines, these latter related to evasion of the host immune response (Cwiklinski et al., 2015a; Liu et al., 2017). Existing data of ESPs is mostly related to non-nuclear factors. However our study predicted that a group of ES proteins from liver flukes may target the host cell nuclei. These proteins should be delivered to host cells through specialized delivery mechanisms such as exosomes or EVs which are vehicles for worms ES proteins transport to host cells (Nawaz et al., 2019). The cargo of EVs from *F. hepatica* and *O. viverrini* have been studied through proteomics approaches and the existence of multiple secretory products have been demonstrated (Cwiklinski et al., 2015b; Chaiyadet et al., 2015; Zakeri et al., 2018). There are differences between the cargo and effect mediated by EVs from *O. viverrini* and *F. hepatica*. Released products from EVs of *O. viverrini* trigger gene expression of cancer related genes and wound healing process genes and further lead to develop a tumorigenic phenotype in human cholangiocytes (Chaiyadet et al., 2015). On the other hand, EVs secreted from *F. hepatica* act not only as immune modulators but also are able to sequestrate triclabendazole from the culture media (Marcilla et al., 2012; de la Torre-Escudero and Robinson, 2017; Murphy et al., 2020; Davis et al., 2020). By applying in silico approaches we identified one polypeptide (A0A074ZQZ9) present in EVs of *O. viverrini* and predicted other 36 that could be found either in ESPs or EVs. Given that secretion and cargo of EVs depends both on biological stage of parasites and on the technique applied, the existence of the nuclear ES proteins here predicted is plausible.

Pathogens that cause cancer are not considered promoters due to its ability to stimulate cell proliferation. This action is performed by some unique factors that interact with host cell proteins, both in cytoplasm and nucleus, thus displaying a direct effect on cell cycle and survival. Of particular interest are those proteins released by infectious agents that cross the nuclear membrane and can interact with nuclear factors and DNA. Those elements may virtually hijack the host cell cycle by controlling critical processes such as cell cycle, apoptosis, survival and response to DNA damage. Our study predicted that *O. viverrini. C. sinensis* and *F. hepatica* have secretory DNA- and RNA-binding proteins such as Homeobox domain-containing proteins, Zinc finger domain proteins, and Cyclophilin E. Similar findings have been reported in bacteria such as *M. hominis* and *C. pneumoniae*, where secretory DNA-binding proteins have been predicted and suggested to have a role in carcinogenesis (Khan et al., 2016a; Alshamsan et al., 2017). In contrast, our findings show that secretory DNA-binding proteins are present in *O. viverrini, C. sinensis* and *F. hepatica* suggesting that it is unlikely the involvement of such proteins in liver fluke-induced carcinogenesis but these may contribute to liver fluke pathogenesis. Actually, cell transformation displayed by *O. viverrini* infection is not only associated with chronic inflammation and proliferation secretory factors that promote cell growth but also with DNA damage such as adducts (Brindley et al., 2015). Other proteins expressed by *O. viverrini* may be able to manipulate some biological process of the host cells by altering certain pathways and molecules both in the membrane and cytoplasm. For instance, thioredoxin, a component of ESP, is a growth factor and apoptosis inhibitor and it might contribute to carcinogenesis (Young et al., 2014; Shi et al., 2020). Similarly, the genesis of *C. sinensis-*induced CCA is also a complex process where certain ES proteins such as cystatin and Oxidoreductase-peroxiredoxin and carbonyl reductase 1 (CBR1) are likely implicated in (Shi et al., 2020). Whether some RNA- and DNA-binding proteins secreted by liver flukes contribute with carcinogenesis or other infection-related features remains unclear.

In summary, we predicted nuclear ESPs of liver flukes by applying an algorithm that is not dependent on presence of NLS which is more suitable given that only 30% of nuclear targeting proteins has NLS (Cokol et al., 2000). The TFIIB-type domain-containing protein of *O. viverrini* and Zinc finger protein 629 of *C. sinensis* may disrupt either replication or transcription process, respectively, in host cells. Further studies are needed to demonstrate whether the predicted polypeptides present in carcinogenic liver flukes participate in cell tumorigenesis.

## Declarations

### Author contribution statement

Claudia Machicado: Conceived and designed the experiments; Performed the experiments; Analyzed and interpreted the data; Contributed reagents, materials, analysis tools or data; Wrote the paper.

Maria Pia Soto: Performed the experiments; Analyzed and interpreted the data; Wrote the paper.

Luis Felipe La Chira, Joel Torres, Carlos Mendoza: Performed the experiments; Analyzed and interpreted the data.

Luis A. Marcos: Conceived and designed the experiments; Analyzed and interpreted the data; Contributed reagents, materials, analysis tools or data; Wrote the paper.

### Funding statement

This research did not receive any specific grant from funding agencies in the public, commercial, or not-for-profit sectors.

### Data availability statement

Data included in article/supplementary material/referenced in article.

### Declaration of interests statement

The authors declare no conflict of interest.

### Additional information

No additional information is available for this paper.
